# Melon Genome Regions Associated with TGR-1551-Derived Resistance to *Cucurbit yellow stunting disorder virus*

**DOI:** 10.3390/ijms21175970

**Published:** 2020-08-19

**Authors:** Ana Pérez-de-Castro, María López-Martín, Cristina Esteras, Ana Garcés-Claver, Francisco Javier Palomares-Ríus, María Belén Picó, María Luisa Gómez-Guillamón

**Affiliations:** 1 Instituto de Conservación y Mejora de la Agrodiversidad Valenciana (COMAV), Universitat Politècnica de València, Camino de Vera, 46022 Valencia, Spain; malom11m@posgrado.upv.es (M.L.-M.); criesgo@upvnet.upv.es (C.E.); mpicosi@btc.upv.es (M.B.P.); 2 Centro de Investigación y Tecnología Agroalimentaria de Aragón, Unidad de Hortofruticultura, Instituto Agroalimentario de Aragón-IA2 (CITA-Universidad de Zaragoza), 50059 Zaragoza, Spain; agarces@cita-aragon.es; 3 Instituto de Hortofruticultura Subtropical y Mediterránea ‘La Mayora’ (IHSM, UMA-CSIC), Algarrobo-Costa, 29760 Málaga, Spain; f.palomares@enzazaden.es (F.J.P.-R.); guillamon@eelm.csic.es (M.L.G.-G.)

**Keywords:** CYSDV, QTLs, molecular markers

## Abstract

*Cucurbit yellow stunting disorder virus* (CYSDV) is one of the main limiting factors of melon cultivation worldwide. To date, no commercial melon cultivars resistant to CYSDV are available. The African accession TGR-1551 is resistant to CYSDV. Two major quantitative trait loci (QTLs) have been previously reported, both located near each other in chromosome 5. With the objective of further mapping the gene or genes responsible of the resistance, a recombinant inbred line (RIL) population derived from the cross between TGR-1551 and the susceptible cultivar ‘Bola de Oro’ was evaluated for resistance to CYSDV in five different assays and genotyped in a genotyping by sequencing (GBS) analysis. The major effect of one of the two QTLs located on chromosome 5 was confirmed in the multienvironment RIL assay and additionally verified through the analysis of three segregating BC_1_S_1_ populations derived from three resistant RILs. Furthermore, progeny test using the offspring of selected BC_3_ plants allowed the narrowing of the candidate interval to a 700 kb region. The SNP markers identified in this work will be useful in marker-assisted selection in the context of introgression of CYSDV resistance in elite cultivars.

## 1. Introduction

The *Cucurbit yellow stunting disorder virus* (CYSDV) is a *Crinivirus* of the family *Closteroviridae* [1,2]. The most important cucurbit crops are infected by this virus, and melon (*Cucumis melo* L.) is one of the species most severely affected.

CYSDV was first reported in the United Arab Emirates [3] and subsequently spread throughout the Middle East and the Mediterranean Basin [4,5,6,7,8,9,10], North and Central America [9,11,12,13], and China [14]. Nowadays, this virus has become a significant threat throughout the cucurbit production regions in the southern United States, Mexico, and Central America [15] and has been reported as the most economically important of the viruses affecting cucurbit production in the southwestern United States [16].

Although initially thought to be restricted to members of the family *Cucurbitaceae*, more recent studies have demonstrated that CYSDV can infect species from at least nine plant families [17].

CYSDV is exclusively transmitted by the sweet potato whitefly, *Bemisia tabaci*, in a semipersistent manner [4]. Although the virus can be transmitted by individual whiteflies, feeding by large numbers of viruliferous insects improves transmission rates [4]. To date, artificial infection methods such as mechanical inoculation or agroinfiltration have not been successful.

The virus can often remain latent for a relatively long period, up to three to four weeks after infection [5]. The infection appears with spotted/mottled symptoms early on, followed by extensive interveinal chlorosis. As with other criniviruses, symptoms are more prominent on older leaves, with younger leaves remaining symptomless. CYSDV infections result in reduced plant vigor [15]. The main damage is produced if the virus infects young plants, which can cause an important yield reduction in terms of fruit number and weight [11,18]. Symptoms in fruits are not obvious, but a decrease in sugar levels in CYSDV-infected plants has also been reported [7,18]. These effects on yield and quality cause important economic losses in many areas.

So far, the main strategy to limit the incidence of vector-borne viral diseases has been the application of insecticides to reduce vector populations combined with selected cultural practices. However, environmental concerns and the ability of the vectors to develop insecticide resistance necessitate the development and deployment of strategies that do not rely on chemicals. Genetic resistance combined with cultural practices could become a viable means to increase yields in crops produced in open fields despite the presence of viruses [19].

Resistance to CYSDV in melon was first reported in the African accession TGR-1551 [18]. Later, the Indian accession PI 313970 was also described as resistant, although this resistance was partial as plants showed late symptoms [20]. Resistance from PI 313970 has been reported as monogenic recessive [21].

TGR-1551 is tolerant to the vector, *Bemisia tabaci* [22], and resistant to the virus itself [18]. The resistance mechanism to the virus in TGR-1551 has been shown to involve a restriction of the virus movement in the vascular system and/or prevention of high levels of virus accumulation [23]. The initial segregation analyses in the family produced from the cross between TGR-1551 and the susceptible Spanish cultivar ‘Piel de Sapo’, suggested a monogenic dominant control of the resistance [18]. Other studies proposed a codominant or more complex nature of the resistance [24]. Subsequent analysis revealed differences in TGR-1551 response to different CYSDV isolates [25,26]. Moreover, the evaluation date has an effect on the CYSDV response; for example, later assessment dates result in high symptom scores in the heterozygotes, thus suggesting a recessive control of the resistance [26]. Recent work suggested that the resistance from TGR-1551 and PI 313970 may be allelic [27].

Presently, there are no commercial cultivars resistant to CYSDV. On one hand, the nature of the exotic resistant germplasm, of poor agronomic value, makes the introgression of resistance into a commercial background difficult. Moreover, phenotyping for resistance in the context of breeding programs is challenging. Besides the bias caused by plants that escape whitefly infection, the difficulty in discriminating among viruses based on symptoms is a characteristic of criniviruses [28]. Yellowing symptoms can also be confused with nutritional disorders or phytotoxicity, among others [5]. As a consequence, differentiation requires detection testing, preferably using sensitive nucleic acid detection methods [28]. Protocols for quantification of CYSDV viral titers based on qRT-PCR are available [29]. The most frequent approach in breeding programs for resistance to CSYDV is the use of controlled inoculations, mediated by *B. tabaci*, accompanied by diagnosis by molecular tests.

The availability of molecular markers for use in marker-assisted selection (MAS) allows avoiding the aforementioned difficulties. In the case of CYSDV, two main quantitative trait loci (QTLs), both located near in chromosome 5, have been reported as linked to resistance derived from TGR-1551 [30,31]. However, further mapping of the gene or genes responsible for the resistance is needed in order to determine markers suitable for MAS.

The objective of this work was to better depict the genetic architecture of the CYSDV resistance derived from TGR-1551, with the aim of identifying molecular markers useful for MAS in breeding programs dedicated to the introgression of this resistance into elite cultivars. To attain this objective, several populations, a recombinant inbred line (RIL) population and derived advanced backcrosses, obtained from an original cross between TGR-1551 and the highly CYSDV-susceptible Spanish cultivar ‘Bola de Oro’ have been genotyped and evaluated for CYSDV resistance.

## 2. Results

### 2.1. Multienvironment Phenotyping for Resistance to CYSDV of the RIL Population

The RIL population (F_7_/F_8_ generations) derived from the cross TGR-1551 × ’Bola de Oro’ (BO) was evaluated for resistance to CYSDV in five different years. Disease assessment was carried out by the evaluation of symptom development in all the assays. Additionally, the estimation of virus titer in plants by qRT-PCR was done in 2012 and 2013 (Supplementary Table S1).

All BO plants showed symptoms in all the assays, whereas TGR-1551 plants remained asymptomatic. Plants of the F_1_ showed slight symptoms, later and less severe than those observed in BO plants. Viral accumulation was not detected in the resistant parent, TGR-1551. The virus titer in BO was either significantly higher than in the F_1_ or similar, depending on the assay (Supplementary Table S1).

Correlations between symptom scores in the different years were moderate to high and highly significant in all cases (values between 0.367 and 0.839, *p* < 0.005 in all comparisons; Supplementary Table S2). Correlations between virus titer values measured by qRT-PCR in the 2012 and 2013 assays were high and highly significant in all comparisons (0.750–0.824, *p* < 0.0001; Table S2). In 2012 and 2013, it was possible to relate symptoms and virus titer. Correlation between symptom score and virus titer was high and highly significant for both years (0.513–0.776, *p* < 0.0001; Supplementary Table S2). The severity of symptoms does not always correlate with virus titer [32]. However, a significant correlation between symptom development and viral accumulation has been reported in melon viral diseases caused by viruses of different families, such as the geminivirus *Tomato leaf curl New Delhi virus* [33] or the potyvirus *Watermelon mosaic virus* [34]. Concretely, in the case of CYSDV, previous studies found a relationship between the time of symptoms appearance and viral accumulation, with delayed development being observed in plants with lower titers [35]. A positive correlation between CYSDV viral titer and whitefly transmission in cucurbit hosts has been reported [35]. Thus, the fact that lower symptoms found in TGR-1551-derived resistant plants correspond to lower viral titers is epidemiologically important in the reduction of inoculum sources in fields.

The effects of genotype (G), environment (E), and the interaction between them (GxE) were explored with data of the 46 coincident RILs included in the 2009, 2010, 2012, and 2013 assays. Genotype, environment, and the interaction between them each had a significant effect (*p* < 0.0001). Although differences among RILs were observed due to the effect of the interaction GxE, symptoms were significantly lower on average in 2009. On the contrary, symptom scores were significantly higher in 2012. *B. tabaci* confined in clip-cages was the inoculation method used in 2009, and plants were kept in pots in a glasshouse once inoculated. It seemed that the cultivation and growing systems led to lower symptom development. Massive inoculation was used in 2010, 2012, and 2013, and plants were grown in a plastic greenhouse. The highest temperatures were registered in 2012 (data not included), which could explain the higher symptom scores observed in this assay.

Resistance to CYSDV in TGR-1551 has been reported as associated with restriction of the virus movement and/or reduction in virus replication [23], which could be limiting the cascade of events associated with symptom induction in TGR-derived resistant plants [36]. Our results show that despite the high correlation between symptoms and viral accumulation, there are symptomless plants in which the virus is detected and, also, plants with symptoms of yellowing that could be attributed to CYSDV in which the virus cannot be detected in the moment of the analysis. The accumulation of the particles and restriction of the virus movement in TGR-1551 has previously been reported [23,27], which could explain the virus titer found in symptomless plants. On the other hand, as previously stated, yellowing symptoms caused by CYSDV are sometimes difficult to visually distinguish, not only from symptoms produced by other criniviruses, but also from nutritional disorders or phytotoxicity [5,28], which can explain the occurrence of symptomatic plants with a negative qRT-PCR. All these factors condition the evaluation of resistance against CYSDV and highlight the need to use efficient disease assessment methodologies in breeding programs. In this work, the availability of evaluation data (both symptoms and virus titer) for most of the RIL lines (several plants per RIL) allowed an accurate classification of each RIL as resistant or susceptible.

### 2.2. QTL Analysis With the RIL Population

**Figure 1:**
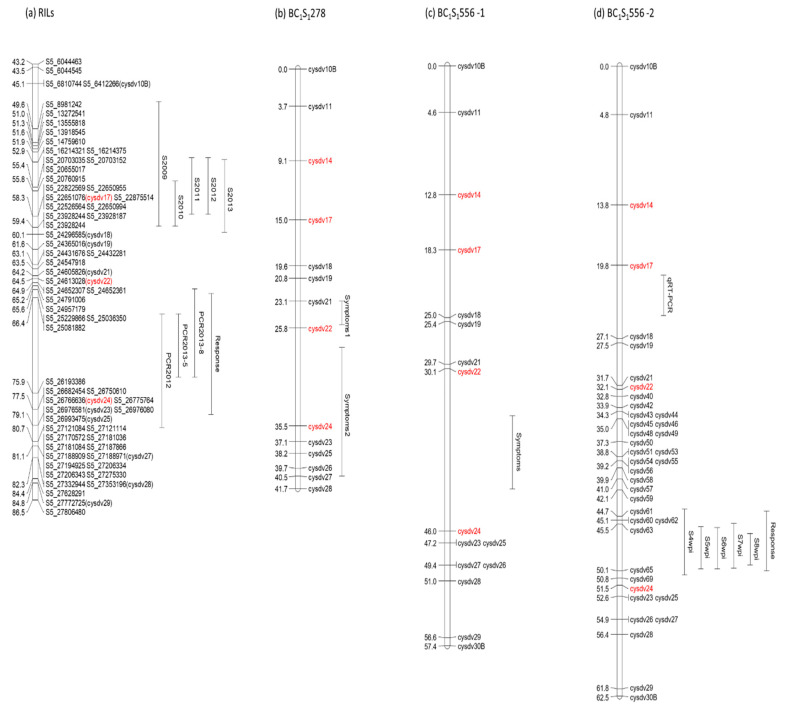
Map of the region of chromosome 5 involved in resistance to *Cucurbit yellow stunting disorder virus* (CYSDV) generated with data from (**a**) the RIL populations genotyped by genotyping by sequencing (GBS), (**b**) the BC_1_S_1_278 population genotyped with the marker set CYSDV1, (**c**) the BC_1_S_1_556 population genotyped with the marker set CYSDV1, and (**d**) the BC_1_S_1_556 population genotyped with the marker sets CYSDV1 and CYSDV2. The QTLs obtained in each of the assays are represented by bars. Markers defining the most important QTLs are indicated in red to facilitate comparison of the different maps. See text and Table 1, Table 2 and Table 3 for description of the traits analyzed.

A QTL analysis was performed using the GBS1 and phenotypic data of the RILs evaluated in each assay. The map used was the SNP map previously constructed with the GBS1 data from the RILs [37]. Chromosome 5 systematically appeared in all the assays as involved in resistance to CYSDV (Table 1). Two genomic regions were identified in this chromosome: one explained variation in symptom severity, while the other explained variation in virus titer detected by qRT-PCR. The QTL associated with symptom severity explained between 27% and 62% of the variance for this trait and, considering the intervals defined for each assay, spanned between 6,810,744 and 24,296,585 bp. For virus titer detected by qRT-PCR, the QTL identified explained between 49% and 53% of the variance, spanning between 24,791,006–27,121,114 bp (Table 1). Both intervals were near a region for which polymorphic markers were not available from the GBS1, of approximately 10 cM and 1.1 Mb (between 66.4 and 75.9 cM, from 25,229,866 to 26,193,386 bp) (Figure 1). This would possibly interfere with the results of the QTL analyses.

As aforementioned, considering evaluation data gathered in all the assays, each RIL was classified as resistant or susceptible. The QTL obtained with this phenotypic value (response in Table 1) explained 44% of the variance, with the interval extending from 24,957,179 to 26,993,475 bp. This interval included the 10 cM region lacking markers (Figure 1), which could account for the size of the interval.

### 2.3. Phenotyping for Resistance to CYSDV and QTL Analysis in Three Selected BC_1_S_1_ Progenies Derived from Selected Resistant RILs

Three of the most resistant and vigorous RILs evaluated in most of the assays, RIL 278, RIL556, and RIL110, were selected to produce segregating generations (Figure S1).

These RILs were backcrossed to the susceptible parent, BO, and one plant from each of the BC_1_ generations was selfed to generate the BC_1_S_1_ populations. The selection of the resistant RILs was done before the GBS1 was available. Genotyping by sequencing (GBS) results of the RILs confirmed later that RIL110 and RIL556 were both homozygous for the TGR allele for the whole chromosome 5 (Table S3). RIL278 was also TGR homozygous for almost the whole chromosome 5, with the exception of the region below 27,628,291, for which it was homozygous for BO alleles.

**Figure 2:**
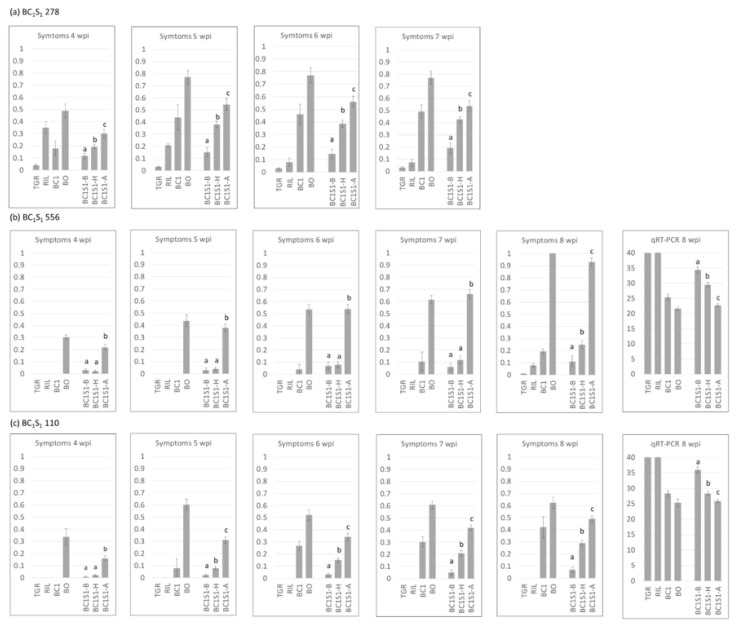
Average symptom score (percentage of leaves showing clear symptoms of CYSDV infection) for different evaluation dates (wpi: weeks post infection) and average Ct (cycle threshold) in the qRT-PCR carried out to detect CYSDV (when available). (**a**) BC_1_S_1_278; (**b**) BC_1_S_1_556; (**c**) BC_1_S_1_110. Genotypes are expressed as follows: TGR: TGR-1551; RIL: selected RIL, RIL278, 556, or 110; BC_1_: first backcross to ’Bola de Oro’ of the selected RIL; BO: ’Bola de Oro’; BC_1_S_1_: selfing progeny from the BC_1_, classified according to the genotype for marker cysdv22 for RIL278 and marker cysdv63 for RILs 556 and 110, where BC_1_S_1_-B is homozygous for TGR-1551 allele, BC_1_S_1_-H is heterozygous, and BC_1_S_1_-A is homozygous for ‘Bola de Oro’ allele. Bars represent standard error. Different letters in the same evaluation date indicate significant differences among genotypes (*p* < 0.05, LSD test).

BC_1_S_1_ plants derived from RIL278 and RIL556 were phenotyped for resistance in 2016. Plants of the susceptible parent BO started showing symptoms from the first evaluation dates, which increased with time (Figure 2). Viral accumulation was confirmed in all plants by qRT-PCR. Some plants of the resistant parent, TGR-1551, showed slight spotting, although it was later confirmed by qRT-PCR that this was not caused by the presence of the virus (Supplementary Table S1). Similarly, the yellowing observed on some evaluation dates in plants of the RILs 278 and 556 could not be attributed to the virus. Along with the parents, some plants of each BC_1_ generation (selected RIL × BO) were evaluated. BC_1_ plants showed milder symptoms than BO at all evaluation dates for both RILs. The first symptoms in the BC_1_ derived from RIL556 appeared delayed with respect to BO. The virus was detected in all BC_1_ plants but at lower levels than in BO.

The first set of markers (derived from the RIL GBS) was designed for its use in the Agena Bioscience genotyping platform (CYSDV1; Supplementary Table S4). This set included markers covering the genomic regions of the two QTLs obtained from the RIL analysis. The QTL analysis was performed with symptom evaluation in the 133 BC_1_S_1_278 plants and the 134 BC_1_S_1_556 plants. (Table 2). A QTL was identified for both families in the region around markers cysdv22 (24,613,028 bp) and cysdv24 (26,766,636 bp), which explained 21% and 58% of the variation in symptom evaluation in both families, respectively (Figure 1, Table 2). These results confirmed the QTL positions found in the RIL populations but supported the need to saturate the region between markers cysdv22 and cysdv24.

For subsequent analyses, the availability of new GBS data (GBS2) allowed the design of molecular markers in the 10 cM region lacking markers in GBS1 (Figure 1) to better delimit the candidate interval. This new set of markers (CYSDV2) was used to genotype the 134 BC_1_S_1_556 plants; the evaluation period in this population extended to 8 weeks post infection (wpi), and virus titer was measured in all plants. To perform the QTL analysis of this population more accurately, a new map of the region was constructed with the genotyping data of the plants with both marker sets, CYSDV1 and CYSDV2, which covered 62.5 cM, corresponding to the region from 6,412,266 to 27,806,568 bp (Figure 1). The new QTL analyses, using this map and the BC_1_S_1_556 population phenotyping results, confirmed the occurrence of a major QTL in this region of chromosome 5 (Table 3). The intervals for the QTL considering the different evaluation dates were overlapping, and altogether covered the region between 25,619,503 and 26,688,074 bp. The closest marker to the LOD peak was cysdv63. Almost the same interval (25,619,503–26,629,653 bp) corresponded to the QTL obtained when assigning a phenotype to each RIL, considering the symptom evaluation and the virus titer (Table 3). In any case, the percentage of the variance explained for the different evaluation dates showed an increasing trend with time (from 33% to 56%). Evaluation 5 (8 wpi) was the date that explained the highest percentage of the variation and had the highest LOD. The interval for the QTL in this evaluation date comprised the region between 25,982,529 and 26,629,653 bp, thus suggesting that this late phenotyping allowed a more accurate characterization of the population. A second QTL associated to the qRT-PCR was identified in the interval between markers cysdv17 and cysdv18 (22,651,076–24,296,585 bp), which explained 44% of the variation for the trait (Table 3).

To evaluate the effect of the symptom-related QTL in both progenies, BC_1_S_1_278 and BC_1_S_1_556 plants were grouped according to their genotype for the closest marker to the higher peak available in each case (cysdv22 for BC_1_S_1_278 and cysdv63 for BC_1_S_1_556). The average symptom score in each evaluation date was calculated for each genotypic class (Figure 2). In the case of BC_1_S_1_556, virus titer was also available. Symptom scores from the first evaluation dates were significantly higher in plants homozygous for the BO allele with respect to the rest of the genotypes in BC_1_S_1_. Symptoms in homozygous plants for the BO allele were slightly lower than in BO, corresponding to a slightly lower virus titer (evaluated in BC_1_S_1_556). Differences between heterozygous BC_1_S_1_ plants and those homozygous for the TGR allele were significant for all evaluation dates in BC_1_S_1_278, but they were only significant at 8 wpi in BC_1_S_1_556. In the case of heterozygote BC_1_S_1_ plants, the average symptom score was similar to those in the corresponding BC_1_, while virus titer (measured in the 556 progenies) was lower in the former. Average scores in plants homozygous for the TGR allele were higher than those observed in the resistant parent, TGR, and in the corresponding RILs (only in the final evaluation date in the case of BC_1_S_1_278), given that, as previously explained, the observed yellowing in the RILs was not attributable to the presence of virus.

To confirm the previous results, BC_1_S_1_ progeny derived from RIL110 were phenotyped for resistance to CYSDV in 2017 (Supplementary Table S1). Percentage of plant infection did not reach 100% in the susceptible control BO at the end of the assay (8 wpi) and remained at levels similar to those obtained in the BC_1_S_1_278 assay, thus suggesting lower inoculum pressure with respect to the BC_1_S_1_556 assay (Figure 2). Similarly, the virus titer detected in the susceptible control in the BC_1_S_1_110 assay was significantly lower at 8 wpi than in the BC_1_S_1_556 assay. Resistant controls in the three assays displayed responses similar to those of TGR-1551, and the resistant RILs remained virus-free. On the contrary, symptom scores in BC_1_110 and BC_1_278 were higher than in BC_1_556, while virus titer was comparable in both assays.

The 146 BC_1_S_1_110 plants were classified according to their genotype for marker cysdv63 (Figure 2). Differences between the three genotypic classes were significant from the second evaluation date to the end of the assay. Symptoms in BC_1_S_1_ plants homozygous for the BO allele were lower than in the assay with family 556, supporting the results obtained in the susceptible control BO. In any case, symptoms in BC_1_S_1_ plants homozygous for the BO allele were slightly lower than in the susceptible parent, BO. Similarly, symptoms in BC_1_S_1_ heterozygous for cysdv63 were lower than in the heterozygous BC_1_ for most evaluation dates. Plants homozygous for the TGR allele showed slight symptoms, corresponding to the detection of virus titer, while TGR-1551 and RIL110 remained asymptomatic and virus-free.

Differences found among the three BC_1_S_1_ populations (all segregating for the TGR-1551 candidate introgression in chromosome 5) could be explained by differences in the genetic background for the rest of the genome. Moreover, as previously stated, there is an important effect of environmental conditions on TGR-1551-derived resistance to CYSDV [27]. The most important differences in this work were found in heterozygous genotypes. These results agree with differences found in previous works, where the F_1_ generation derived from the initial cross TGR-155 × BO behaved either as resistant [18] or susceptible [27]. The heterozygous plants in the three segregating BC_1_S_1_ populations analyzed here showed (for the percentage of infection at the later evaluation date, 8 wpi) an intermediate response between TGR-1551 and BO. However, the evolution in these heterozygous plants from the segregating generations supported the previous findings that established later evaluation dates as more representative [27], as percentage of infection on the initial sampling dates did not differ from that of plants that were homozygous for the TGR-1551 allele in some of the assays.

### 2.4. Progeny Test Narrowing the Interval of the Major QTL and Confirmation of Its Effect in Advanced Backcross Selfing Populations

To complement the analysis of the RIL and BC_1_S_1_ RIL derived populations, and to evaluate the effect of the introgression in plants with BO genetic background, 200 plants of the BC_3_ (TGR-1551 × BO derived) population were genotyped with marker set CYSDV1. Fifty-two plants were selected for the presence of different introgressions in the candidate region of chromosome 5. These plants were additionally genotyped with a set of 124 markers evenly distributed throughout the genome. As an average, 82% of the genome in the 52 plants corresponded to regions homozygous for BO alleles. Fifteen BC3 plants were selected to obtain the BC_3_S_1_ progenies. The selection was based on the presence of different introgressions in the candidate region of chromosome 5 (Table 4) and prioritized a high percentage of the BO genetic background for the rest of the genome.

The phenotyping/genotyping results of these offspring were compatible with the interval for the major QTL obtained in the analyses of the BC_1_S_1_556 plants (Table 4, Supplementary Table S1). Recombinants in the region allowed the delimitation of the candidate region. The interval obtained considering the different evaluation dates in BC_1_S_1_556 plants covered the region between 25,619,503 and 26,688,074 bp. Segregation among BC_3_S_1_ plants derived from BC_3_ 166 and 198, and susceptibility of descendants from BC_3_ 105, 141, and 146, confirmed the location of the resistance gene between markers cysdv63 and cysdv65 (25,982,529–26,629,653 bp). All BC_3_ progenies segregated according to the presence of the resistance gene(s) in the region between markers cysdv63 and cysdv65, producing susceptible selfing progenies (24, 28, 37, 78, 96, 105, 141, and 146), or segregating offspring (15, 19, 64, 95, 159, 166, and 198) (Table 4). The proposed interval explained the phenotype of the RILs; excluding recombinants in the region, there was cosegregation between the phenotype and the genotype in 87% of the homozygous lines for the rest of the RILs (Table S1).

The candidate interval for the major CYSDV resistance QTL obtained in this work (25,982,529–26,629,653 bp) contains 57 predicted genes, 51 of them annotated (Table S5). Several of the annotated genes in this region have resistance-related functions. In fact, the candidate interval overlaps with a 760 kb region with the highest concentration of resistance genes in the melon genome [38]. This region has been shown to be highly polymorphic at the intra- and interspecific levels, thus explaining differences in resistance found in different melon genotypes [38]. The interval proposed here contains three genes annotated as ‘disease resistance protein’ (MELO3C031332.2, MELO3C004320.2, and MELO3C031556.2), as well as related-resistance genes such as a receptor-like cytosolic serine/threonine-protein kinase (MELO3C004315.2) or two nucleotide-binding site–leucine-rich repeat proteins (MELO3C004319.2 and MELO3C031325.2). The most frequent class was the TMV resistance protein N-like (12 of the annotated genes in the candidate interval), a TIR-NBS-LRR gene having homology with resistance genes [38]. Moreover, this region includes the virus aphid transmission resistance gene (*Vat*) [38], carried by TGR-1551 [39]. The major QTL for resistance to powdery mildew caused by *Podosphaera xanthii* (Castagne) U. Braun & N. Shishkoff races 1, 2, and 5 derived from the resistance source used here, TGR-1551, has also previously been mapped to this region [40]. This will be an advantage in the breeding program for the introgression of TGR-1551 resistance to CYSDV, *Aphis gosypii*, or powdery mildew, given that the three resistances would be transferred simultaneously. In fact, the Yellow Canary breeding melon line ‘Carmen’, derived by backcrossing from the initial cross between TGR-1551 and ‘Bola de Oro’, confirmed this instance [31]. Although resistance to CYSDV was the only selection character used in the backcrossing program, the line ‘Carmen’ resulted in also being highly resistant to powdery mildew and *Aphis gosypii*. Furthermore, quality was assessed in ‘Carmen’; despite carrying the introgression associated with the resistances, its commercial quality was confirmed. TGR-1551 is also resistant to *Watermelon mosaic virus* (WMV), and molecular markers tightly linked to the resistance QTL with a major effect on chromosome 11 have been developed [37]. Resistance to WMV can also be introgressed into elite cultivars in breeding programs with TGR-1551 as a donor parent. Apart from a major QTL of chromosome 11, a minor QTL also associated with resistance to WMV derived from this source is located in this same region of chromosome 5 [37].

Resistance to CYSDV in cucumber (*Cucumis sativus* L.) has been reported in accession PI 250147, characterized by the absence of symptoms [41]. One of the QTLs, which explained the highest percentage of this resistance, seemed to be linked in the repulsion phase to two loci conferring resistance to powdery mildew, derived from different sources. These resistances have been mapped to linkage group 5, in a region syntenic to melon chromosomes 9 and 10. A method to introgress resistance to CYSDV in melon chromosome 9 from an unknown source has been patented [42], but the region is not syntenic with the CYSDV-resistance region in cucumber.

The region of the melon genome interval containing the cluster of putative resistance genes has been studied in detail to analyze the genomic variability [43] in a study that confirmed the difficulty in obtaining an accurate and complete sequence by NGS due to the multiple, highly similar genes clustered in a relatively short region. This may have been the cause of the lack of markers in this region in the initial map developed from the GBS of the RIL populations. In any case, the availability of the subsequent GBS data allowed the identification of markers polymorphic between BO and TGR-1551 to design the marker set CYSDV2, which led to the narrowing of the candidate interval for the TGR-1551-derived resistance. Markers in both sets, CYSDV1 and CYSDV2, were initially implemented for their use in the Agena Bioscience genotyping platform. Some of the markers of both sets have been adapted for their analysis by high-resolution melting (HRM). Concretely, the HRM protocol for markers in the candidate regions has been set (Supplementary Table S4), which allows the efficient identification of the polymorphism between the BO and the TGR alleles by PCR-based markers. The availability of this type of marker, suitable for MAS in the context of breeding programs, is of special interest in the case of introgression of resistance to CYSDV. As previously stated, the yellowing symptoms caused by crinivirus are undistinguishable. Similar symptoms are caused by different criniviruses [28], and these symptoms are commonly confused with the effects of nutritional disorders or phytotoxicity [5]. Thus, the use of MAS is an effective method to circumvent these difficulties.

The analysis of different TGR-1551-derived generations allowed the narrowing of the candidate interval of resistance to CYSDV to a 700 kb region in chromosome 5. This resulted in the availability of molecular markers tightly linked to the resistance. The PCR-based markers developed here are an efficient resource for use in TGR-1551-derived resistance introgression in commercial melons. The breeding program for the introgression of TGR-1551-derived resistance to CYSDV, WMV, and powdery mildew in ‘Bola de Oro’ and ‘Piel de Sapo’ backgrounds has already been initiated. Future work will include further analysis in advanced backcrossed progenies with the purpose of better understanding the effect of the minor QTL obtained in some of the analyses in the region of markers cysdv14 and cysdv17. The linkage between this region and the region of the major QTL limits the availability of segregant generations appropriate to discriminate against the effect of this minor QTL. Moreover, RNAseq analysis will be carried out in order to identify differentially expressed genes in the candidate regions.

## 3. Materials and Methods

### 3.1. Plant Material

The following populations obtained from an original cross between the resistant melon line TGR-1551 and the susceptible Spanish melon cultivar ‘Bola de Oro’ (BO) have been evaluated against CYSDV (Supplementary Figure S1):

1. An RIL population F_7_/F_8_, developed by the single seed descent method [30];
2. Three BC_1_S_1_ progenies derived from crosses between three resistant RILs and BO;
3. Fifteen BC_3_S_1_ progenies derived from 15 BC_3_ (× BO) selected for their genotype in the candidate region.

Each RIL population (3–4 plants/RIL), together with its parental genotypes and their F_1_, was evaluated in up to five different assays (number of lines in parentheses): Spring 2009 (86), Spring 2010 (101), Spring 2011 (73), Summer 2012 (88), and Summer 2013 (121). Not all RILs were evaluated in all the assays due to seed availability. The different backcross and selfing populations were phenotyped in additional screening assays according to the methodology described below.

### 3.2. Inoculation Method

Healthy whitefly colonies (*Bemisia tabaci*) reared on plants of the susceptible Spanish melon accession ‘ANC-57’ were used in all the inoculations and assays. Controlled CYSDV-infected plants of this accession showing clear and typical symptoms of CYSDV were used as inoculum source of the virus in all the inoculations.

RIL assays conducted in 2009 and 2011 were carried out by using *B. tabaci* confined in clip-cages following the methodology developed by [4], in which 60 whiteflies were fed on the inoculum source for 48 h (acquisition time) and then transferred to young plants (1–2 true leaves) to feed for 48 h (transmission time). Once inoculated, plants were sprayed with imidacloprid to kill the whiteflies and transplanted to pots in the glasshouse. Assays conducted in 2010, 2012, and 2013 were carried out by massive inoculation. Plants of each RIL at the stage of 2–3 true leaves were transplanted to a plastic greenhouse where they were randomly distributed interspersed with infected BO plants. One week after transplanting, viruliferous whiteflies were released in the plot to enhance virus transmission. In this case, any spraying against whiteflies was done over the course of the experiment.

The clip-cage method was used in the inoculation of subsequent generations.

### 3.3. Disease Assessment

Virus symptoms were visually assessed once a week over a one-month period (2009, 2010, and 2011) or over a two-month period (2012 and 2013). The symptom scoring in Spring 2009 and Spring 2010 was based on a visual scale of the number of leaves with viral symptoms, ranging from 0 (no symptoms) to 9 (nine leaves with clear virus symptoms). In Spring 2011 and Summer 2012, the score was based on a visual scale of virus symptoms, ranging from 0 (no symptoms) to 5 (almost the entire plant with clear symptoms). In Summer 2013, the scoring was based on the percentage of leaves showing typical CYSDV symptoms of infection (number of leaves affected/total leaf number).

In the trials of 2012 and 2013, the third leaf from the plant apex of each plant was sampled 14 days after virus inoculation (2012) or each week (2013) to estimate the virus titer in the plants by qRT-PCR, following [29].

During the Spring 2016 and Spring 2017 assays, three BC_1_S_1_ progenies derived from crosses between three resistant RILs (278, 556, and 110) and BO were evaluated. Virus inoculations were carried out by using *B. tabaci* confined in clip-cages as explained above. Once inoculated, plants were transplanted to the greenhouse. Evaluation of CYSDV was based on the percentage of leaves showing typical CYSDV symptoms of infection (number of leaves affected/total leaf number). qRT-PCR analyses to estimate the virus titer in the plants were carried out every week (2016) or every two weeks (2017).

Additionally, in 2019, BC_3_S_1_ progenies (12 to 20 plants each) derived from 15 BC_3_ plants selected according to their genotype in the candidate region of chromosome 5 were inoculated with CYSDV using the clip-cage method. Then, plants were transferred to the greenhouse and evaluated by symptoms and qRT-PCR as described for the RIL110 BC_1_S_1_ population.

### 3.4. Statistical Analyses

Analysis of variance (ANOVA) using data from four of the RIL evaluations was carried out. Correlations between pairs of environments were estimated by using the Pearson correlation coefficient. Comparisons of pairs of means in different assays were performed using the LSD test with a probability level of *p* < 0.05. Statistical analyses were conducted using Statgraphics Centurion XVI.I software (StatPoint Technologies, Inc., Warrenton, VA, USA).

### 3.5. Markers and Genotyping Methods

Total DNA was extracted from young leaves following the method described by [44] with minor modifications [45]. DNA concentration was measured using spectrophotometry in a Nanodrop ND-1000 Spectrophotometer v.3.5 (LabTech International, Heathfield, UK) and adjusted to 10 ng/μL for the genotyping analysis.

The SNPs used in this study were designed from data originating from different GBS analysis. The whole RIL population (148 RILs), both parents (BO and TGR-1551) and their F_1_, constituted the plant materials for the first GBS (GBS1 assay). A total of 16 SNPs, located on the candidate region on chromosome 5, were selected from this GBS and constituted the first set of markers (CYSDV1; Supplementary Table S4). Preferably, markers were selected that corresponded to those included in the map used in the analysis. When necessary to evenly cover the region, markers were chosen among the markers discarded for the map because of segregation distortion (only two markers). An additional GBS experiment, GBS2, conducted to perform genetic diversity studies (including BO and TGR-1551, among many other genotypes), was also used as a source for 24 extra SNPs in order to increase the resolution of the candidate region (marker set CYSDV2; Supplementary Table S4). All the SNPs were implemented for their use in the Agena Bioscience platform (https://agenabio.com/products/massarray-system/); use of this platform was carried out in the Epigenetic and Genotyping unit of the University of Valencia, Unitat Central d´Investigació en Medicina (UCIM), Spain. Marker set CYSDV1 was used to genotype the BC_1_S_1_, BC_3_, and BC_3_S_1_ generations. Generations BC_1_S_1_556 and BC_3_S_1_ were also genotyped with marker set CYSDV2. BC_3_ plants were genotyped with an existing panel of 124 SNPs evenly distributed throughout the genome, also implemented in the Agena Bioscience platform. This SNP set had been previously validated in populations derived from ibericus × acidulus melon crosses [45,46,47]. Some of the markers in CYSDV1 and CYSDV2 sets have been adapted to a PCR-based protocol for their analysis by high-resolution melting (HRM) (Supplementary Table S4).

### 3.6. QTL Analyses

QTL analysis was performed using the genotyping results of GBS1 and RIL CYSDV phenotypes. Phenotypic data used were the percentage of infection at the end of each assay and qRT-PCR values, when available. Moreover, a final phenotypic value was assigned to each RIL, considering the five evaluations, so that each RIL was classified as resistant or susceptible. First, the Kruskal–Wallis nonparametric test was used for QTL detection (MapQTL version 4.1 software) [48]. In addition, a composite interval mapping (CIM) approach was performed [49], using a window size of 15 cM and five cofactors (Windows QTL Cartographer v.2.5-009) [50]. For qualitative traits (response, where plants were classified as either susceptible or resistant), qGene v.4.4.0 was the software used [51]. The LOD threshold was determined by a permutation test (1000 cycles). Loci detected by both Kruskal–Wallis and CIM methods were considered robust QTLs. QTL interval was defined as a 2-LOD drop from the peak. The phenotypic effect, expressed as the percentage of phenotypic variance explained, R^2^, and the additive (when possible) and dominance effects were estimated for each QTL. The map used in the QTLs analyses for the RIL population was that constructed with the GBS data of the RILs [37].

Additional QTL analyses were performed with BC_1_S_1_ progenies from RILs 278 and 556. BC_1_S_1_ progenies from RIL 278 were genotyped with a set of SNP markers selected from the GBS of the RILs (CYSDV1), covering the candidate region in chromosome 5. A new set of markers (CYSDV2) that saturated the candidate region was designed; it was used, together with CYSDV1, to genotype the BC_1_S_1_ progenies from RIL 556. A new map of the region was constructed for each of the progenies, and these maps were used for the respective QTL analyses. The software used was MAPMAKER 3.0 [52]. The map was generated using the Kosambi map function.

## 4. Conclusions

No commercial varieties resistant to CYSDV are presently available. The African accession TGR-1551 has been reported as resistant to *Bemisia tabaci* and to the virus itself. The work reported has allowed the narrowing of the candidate interval for the major QTL associated with resistance to CYSDV derived from TGR-1551 to a region of approximately 700 kb. The SNP markers provided here are useful in marker-assisted selection (MAS) in breeding programs aimed at the introgression of CYSDV resistance. CYSDV is exclusively transmitted by its insect vector, *Bemisia tabaci*. Moreover, yellowing symptoms caused by CYSDV are difficult to distinguish from symptoms caused by other viruses, and yellowing provoked by crinivirus is easily confused with the effects of nutritional disorders, phytotoxicity, or other causes. Thus, the availability of markers for MAS in the context of melon breeding programs is essential for accelerating the introgression of resistance to CYSDV into elite cultivars.

## Figures and Tables

**Table 1:** Quantitative trait loci (QTLs) identified in chromosome 5 in the different assays with the recombinant inbred line (RIL) population derived from the cross between TGR-1551 and the cultivar ‘Bola de Oro’, phenotyped for resistance to *Cucurbit yellow stunting disorder virus* and genotyped by sequencing.

Trait ^1^	Interval ^2^	Nearest Marker ^3^	Kruskal–Wallis		Composite Interval Mapping	
			Mean BO ^4^	Mean TGR ^5^	LOD ^6^	R ^2,7^
S2009 (Scale 0–9)	46.9–59.3 cM 6,810,744–23,928,244 bp	S5_16214321	4.93	2.65	6.4	0.27
S2010 (Scale 0–9)	54.8–59.3 cM 16,214,321–23,928,244 bp	S5_22526564	6.70	2.09	18.5	0.62
S2011 (Scale 0–9)	52.5–58.1 cM 14,759,610–22,875,514 bp	S5_16214375	6.16	0.71	17.9	0.47
S2012 (Scale 0–5)	52.5–58.1 cM 14,759,610–22,875,514 bp	S5_16214375	4.26	1.89	12.3	0.34
S2013 (% infection)	52.7–59.9 cM 14,759,610–24,296,585 bp	S5_20703035	43.26	19.27	10.5	0.30
qRT-PCR2012	68.8–79.3 cM 25,036,350–27,121,114 bp	S5_26193386	28.26	35.17	16.1	0.49
qRT-PCR2013-5	68.0–74.3 cM 25,036,35–26,193,386 bp	S5_26193386	26.88	35.06	19.9	0.53
qRT-PCR2013-8	65.5–74.3 cM 24,791,006–26,193,386 bp	S5_25229866	26.09	33.08	15.1	0.51
Response	66-78 cM 24,957,179–26,993,475 bp	S5_25229866	0.94	0.33	12.7	0.44

^1^ Trait: symptom evaluation for the five assays (S2009, S2010, S2011, S2012, and S2013; scale for evaluation is indicated for each assay), qRT-PCR virus titer in 2012 (qRT-PCR2012) and 2013 (the evaluations took place 5 weeks post inoculation for qRT-PCR2013-5 and 8 weeks post inoculation for qRT-PCR2013-8), and response (classification of each RIL as resistant or susceptible considering evaluation data gathered in all the assays). See Section 3 for details. ^2^ Interval position of the putative QTL on the genetic and physical maps according to an LOD drop of 2. The physical position (v3.6.1) is defined by the position of the markers flanking the QTL interval. ^3^ Closest marker to the LOD peak. Significance level in the Kruskal–Wallis test was 0.0001 for all the markers. ^4^ Mean of the genetic class TGR-1551 for the corresponding marker. ^5^ Mean of the genetic class ‘Bola de Oro’ for the corresponding marker. ^6^ Higher logarithm of the odds score. ^7^ Percentage of phenotypic variance explained by the QTL.

**Table 2:** Quantitative trait loci (QTLs) identified in chromosome 5 in BC_1_S_1_ progenies of RILs 278 and 556 derived from the cross between TGR-1551 and the cultivar ‘Bola de Oro’, phenotyped for resistance to *Cucurbit yellow stunting disorder virus* and genotyped with markers set CYSDV1 (see text for description).

Trait ^1^	Interval ^2^	Nearest Marker ^3^	Composite Interval Mapping	
			LOD ^4^	R ^2,5^
Symptoms 278	23.1–25.4 cM 24,605,826–24,613,028 bp	cysdv22	6.8	0.21
	27.7–40.5 cM 24,613,028–27,188,971 bp	cysdv24	4.5	0.12
Symptoms 556	40.0–48.0 cM 24,613,028–26,993,475 bp	cysdv22	25.5	0.58

^1^ Trait: Symptom evaluation in the different progenies, BC_1_S_1_278 (Symptoms 278) and BC_1_S_1_556 (Symptoms 556). ^2^ Interval position of the putative QTL on the genetic and physical maps according to a LOD drop of 2. The physical position (v.3.6.1) is defined by the position of the markers flanking the QTL interval. ^3^ Closest marker to the LOD peak. Significance level in the Kruskal–Wallis test was 0.0001 for all the markers. ^4^ Higher logarithm of the odds score. ^5^ Percentage of phenotypic variance explained by the QTL.

**Table 3:** Quantitative trait loci (QTLs) identified in chromosome 5 in BC_1_S_1_ progenies of RIL 556 derived from the cross between TGR-1551 and the cultivar ‘Bola de Oro’, phenotyped for resistance to *Cucurbit yellow stunting disorder virus* and genotyped with marker sets CYSDV1 and CYSDV2 (see text for description).

Trait ^1^	Interval ^2^	Nearest Marker ^3^	Composite Interval Mapping	
			LOD ^4^	R ^2,5^
S4wpi	44.0–50.5 cM 25,619,503–26,688,074 bp	cysdv63	14.8	0.33
S5wpi	45.7–49.9 cM 25,982,529–26,629,653 bp	cysdv63	19.9	0.39
S6wpi	45.8–49.9 cM 25,982,529–26,629,653 bp	cysdv63	19.6	0.39
S7wpi	45.4–49.8 cM 25,943,991–26,629,653 bp	cysdv63	17.6	0.39
S8wpi	46.4–49.5 cM 25,982,529–26,629,653 bp	cysdv63	33.1	0.56
qRT-PCR	20.8–24.8 cM 22,651,076–24,296,585 bp	cysdv17	20.4	0.44
Response	44.2–50.1 cM 25,619,503–26,629,653 bp	cysdv63	16.0	0.26

^1^ Trait: Symptom: evaluation on different dates, indicated as S + number of wpi (weeks post inoculation). qRT-PCR: Ct value for the qRT-PCR at 8 wpi. Response: classification of each plant as resistant, moderately resistant, moderately susceptible, or susceptible, considering symptom evaluation and qRT-PCR Ct value. ^2^ Interval position of the putative QTL on the genetic and physical maps according to an LOD drop of 2. The physical position (v.3.6.1) is defined by the position of the markers flanking the QTL interval. ^3^ Closest marker to the LOD peak. Significance level in the Kruskal–Wallis test was 0.0001 for all the markers. ^4^ Higher logarithm of the odds score. ^5^ Percentage of phenotypic variance explained by the QTL.

**Table 4:** Genotype for SNPs in marker sets CYSDV1 and CYSDV2 for the BC_3_ plants selected to evaluate their descendants (A: homozygous for ‘Bola de Oro’ allele; H: heterozygous). The phenotype of the progenies is indicated (SU: susceptible; SE: segregating).

Marker	Position (bp)	15	19	64	95	159	166	198	24	28	37	78	96	105	141	146
cysdv10B	6,412,266	A	A	H	A	H	H	A	H	H	A	A	A	A	A	A
cysdv11	9,593,263	A	A	H	A	H	H	A	H	H	A	A	A	A	A	A
cysdv14	17,265,147	A	A	H	A	H	H	A	H	H	A	A	A	A	A	A
cysdv17	22,651,076	A	A	H	A	H	H	A	H	H	A	A	A	A	A	A
cysdv18	24,296,585	A	H	H	A	H	H	A	A	H	A	A	A	A	A	A
cysdv19	24,365,016	A	H	H	A	H	H	A	A	H	A	A	A	A	A	A
cysdv21	24,605,826	A	H	H	A	H	H	A	A	H	A	A	A	A	H	H
cysdv22	24,613,028	A	H	H	A	H	H	A	A	H	A	A	A	A	H	H
cysdv40	24,652,307	A	H	H	A	H	H	A	A	H	A	A	A	A	H	H
cysdv42	24,792,185	A	H	H	A	H	H	A	A	A	A	A	A	A	H	H
cysdv43	24,864,545	A	H	H	A	H	H	A	A	A	A	A	A	A	H	H
cysdv44	24,890,589	A	H	H	A	H	H	A	A	A	A	A	A	A	H	H
cysdv45	24,945,626	A	H	H	A	H	H	A	A	A	A	A	A	A	H	H
cysdv46	24,962,187	A	H	H	A	H	H	A	A	A	A	A	A	A	H	H
cysdv48	25,026,788	A	H	H	A	H	H	A	A	A	A	A	A	A	H	H
cysdv49	25,027,045	A	H	H	A	H	H	A	A	A	A	A	A	A	H	H
cysdv50	25,236,105	H	H	H	A	H	H	A	A	A	A	A	A	A	H	H
cysdv51	25,314,484	H	H	H	H	H	H	A	A	A	A	A	A	A	H	H
cysdv53	25,326,351	H	H	H	H	H	H	A	A	A	A	A	A	A	H	H
cysdv54	25,392,541	H	H	H	H	H	H	A	A	A	A	A	A	A	H	H
cysdv55	25,392,903	H	H	H	H	H	H	A	A	A	A	A	A	A	H	H
cysdv56	25,415,551	H	H	H	H	H	H	A	A	A	A	A	A	A	H	H
cysdv57	25,526,168	H	H	H	H	H	H	A	A	A	A	A	A	A	H	H
cysdv58	25,540,372	H	H	H	H	H	H	A	A	A	A	A	A	A	H	H
cysdv59	25,619,503	H	H	H	H	H	H	A	A	A	A	A	A	A	H	H
cysdv60	25,943,991	H	H	H	H	H	H	A	A	A	A	A	A	A	H	H
cysdv61	25,956,650	H	H	H	H	H	H	A	A	A	A	A	A	A	H	H
cysdv62	25,975,889	H	H	H	H	H	H	A	A	A	A	A	A	A	H	H
cysdv63	25,982,529	H	H	H	H	H	H	A	A	A	A	A	A	A	H	H
cysdv65	26,629,653	H	H	H	H	H	A	H	A	A	A	A	A	H	A	A
cysdv69	26,688,074	H	H	A	H	H	A	H	A	A	A	A	A	H	A	A
cysdv24	26,766,636	H	H	A	H	H	A	H	A	A	A	A	A	H	A	A
cysdv23	26,976,581	H	H	A	H	H	A	H	A	A	A	A	A	H	A	A
cysdv25	26,993,475	H	H	A	H	H	A	H	A	A	A	A	A	H	A	A
cysdv26	27,170,637	H	H	A	H	H	A	H	A	A	A	A	A	H	A	A
cysdv27	27,188,971	H	H	A	H	H	A	H	A	A	A	A	A	H	A	A
cysdv28	27,353,196	H	H	A	H	H	A	H	A	A	A	A	A	H	A	A
cysdv29	27,772,725	H	H	A	H	H	A	A	A	A	A	A	A	H	A	A
cysdv30B	27,806,568	H	H	A	H	H	A	H	A	A	A	A	A	H	A	A
Phenotype		SE	SE	SE	SE	SE	SE	SE	SU	SU	SU	SU	SU	SU	SU	SU

## Supplementary Materials

Supplementary materials can be found at https://www.mdpi.com/1422-0067/21/17/5970/s1. Figure S1. Generations used and experiments carried out. Table S1. Symptom evaluation and qRT-PCR result after inoculation with CYSDV of the RIL population and advanced derived backcrosses. Table S2. Correlations between parameters used for disease assessment (symptom scores and qRT-PCR Ct values) in the RILs population in the different assays. Table S3. Genotype of RILs 110, 278 and 556 for the markers in GBS 1. Table S4. Marker sets (CYSDV1 and CYSDV2) used in genotyping of progenies. Table S5. Gene loci in the region of the candidate interval for the major QTL for resistance to CYSDV derived from TGR-1551.
