# Supplementary material for: Melon Genome Regions Associated with TGR-1551-Derived Resistance to Cucurbit yellow stunting disorder virus
Source: Int J Mol Sci. 2020 Aug 19;21(17):5970. doi: 10.3390/ijms21175970 (PMC7504372; doi:10.3390/ijms21175970)
Supplement: Supplementary file 1 [file ijms-21-05970-s001.zip › Supplementary_figure_S1.pptx]

## Slide 1
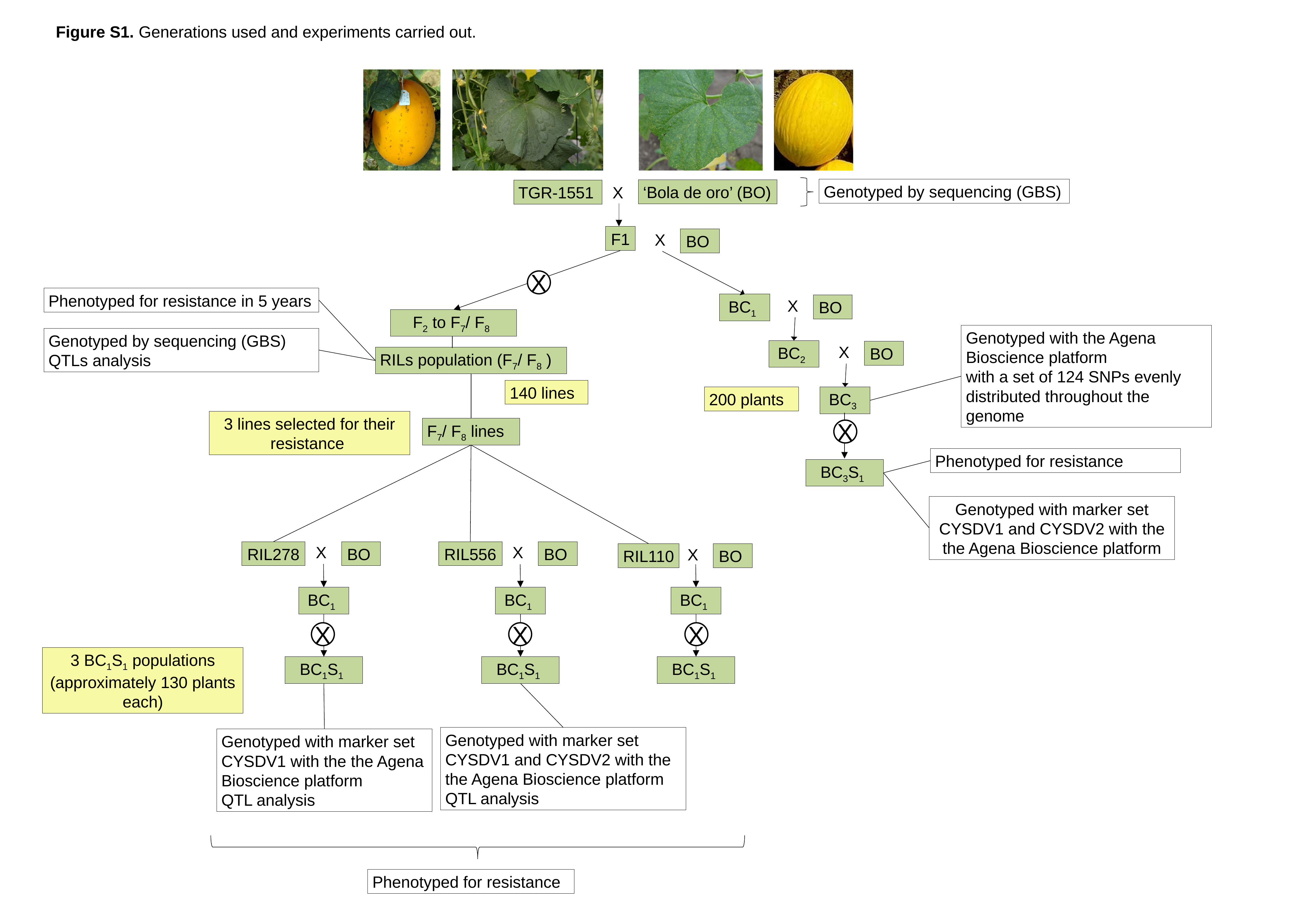

Figure S1. Generations used and experiments carried out.
Genotyped by sequencing (GBS)
‘Bola de oro’ (BO)
TGR-1551
X
F1
X
BO
X
Phenotyped for resistance in 5 years
X
BC1
BO
F2 to F7/ F8
Genotyped with the Agena Bioscience platform
with a set of 124 SNPs evenly distributed throughout the genome
Genotyped by sequencing (GBS)
QTLs analysis
X
BC2
BO
RILs population (F7/ F8 )
140 lines
200 plants
BC3
3 lines selected for their resistance
X
F7/ F8 lines
Phenotyped for resistance
BC3S1
Genotyped with marker set CYSDV1 and CYSDV2 with the the Agena Bioscience platform
X
X
RIL278
BO
RIL556
BO
X
RIL110
BO
BC1
BC1
BC1
X
X
X
3 BC1S1 populations (approximately 130 plants each)
BC1S1
BC1S1
BC1S1
Genotyped with marker set CYSDV1 and CYSDV2 with the the Agena Bioscience platform
QTL analysis
Genotyped with marker set CYSDV1 with the the Agena Bioscience platform
QTL analysis
Phenotyped for resistance
